# SlCV affects starch metabolism by regulating SlBAM3 stability under low night temperature stress in tomatoes

**DOI:** 10.1093/hr/uhaf233

**Published:** 2025-09-03

**Authors:** Jiazhi Lu, Yu Chen, Tianyi Zhang, Feng Wang, Mingfang Qi, Tianlai Li, Yufeng Liu

**Affiliations:** The Modern Facilities Horticultural Engineering Technology Center, Shenyang Agricultural University, Shenyang 110866, China; The Key Laboratory of Protected Horticulture, Ministry of Education, Shenyang 110866, China; Yazhouwan National Laboratory, Sanya 572024, China; The Modern Facilities Horticultural Engineering Technology Center, Shenyang Agricultural University, Shenyang 110866, China; The Key Laboratory of Protected Horticulture, Ministry of Education, Shenyang 110866, China; Inner Mongolia Academy of Agricultural and Animal Husbandry Sciences, Hohhot 010031, China; The Modern Facilities Horticultural Engineering Technology Center, Shenyang Agricultural University, Shenyang 110866, China; The Key Laboratory of Protected Horticulture, Ministry of Education, Shenyang 110866, China; Peking University Institute of Advanced Agricultural Sciences, Shandong Laboratory of Advanced Agriculture Sciences at Weifang, Weifang 261325, China; The Modern Facilities Horticultural Engineering Technology Center, Shenyang Agricultural University, Shenyang 110866, China; The Key Laboratory of Protected Horticulture, Ministry of Education, Shenyang 110866, China; The Modern Facilities Horticultural Engineering Technology Center, Shenyang Agricultural University, Shenyang 110866, China; The Key Laboratory of Protected Horticulture, Ministry of Education, Shenyang 110866, China; The Modern Facilities Horticultural Engineering Technology Center, Shenyang Agricultural University, Shenyang 110866, China; The Key Laboratory of Protected Horticulture, Ministry of Education, Shenyang 110866, China; The Modern Facilities Horticultural Engineering Technology Center, Shenyang Agricultural University, Shenyang 110866, China; The Key Laboratory of Protected Horticulture, Ministry of Education, Shenyang 110866, China

## Abstract

Nocturnal starch remobilization is critical for plant carbon allocation and stress adaptation. While *β*-amylase 3 (BAM3) serves as the primary catalyst for starch degradation at night, its regulation mechanisms under stress remain to be fully characterized. The chloroplast vesiculation (CV) protein is crucial for maintaining chloroplast homeostasis during stress conditions, though its potential involvement in starch metabolic processes remains unexplored. Herein, we show that low night temperature (LNT) stress induces starch accumulation in tomato leaves, with *SlCV* overexpression exacerbating this phenotype and compromising LNT tolerance, whereas *SlCV* silencing promotes starch catabolism. RNA-seq and metabolome analyses detected lower levels of starch metabolites and amylase activity in *SlCV* overexpression plants. Strikingly, we have confirmed the physical interaction between SlCV and SlBAM3, and *SlCV* overexpression significantly accelerated the degradation of SlBAM3 under LNT stress, while *SlCV* knockout enhanced the stability of SlBAM3. Genetic validation confirmed that *SlBAM3*-silenced plants accumulate excessive starch and exhibit LNT-sensitive phenotypes, and *SlBAM3* overexpression enhances cold tolerance. Furthermore, SlBAM3 complementation rescues the starch overaccumulation and LNT hypersensitivity of *SlCV* overexpression plants. These results elucidate the regulatory mechanism of starch metabolism mediated by SlCV and associated with SlBAM3 protein stability, providing novel insights into the starch metabolic pathway under cold stress.

## Introduction

Tomato (*Solanum lycopersicum*), as a globally cultivated crop across tropical, subtropical, and temperate regions, exhibits particular vulnerability to low night temperature (LNT) stress during the winter and spring seasons of cultivation. Cold stress disrupts nearly all physiological processes in plants, particularly photosynthetic efficiency that governs source-sink allocation dynamics [1]. Starch, the primary carbon reserve in photoautotrophic organs, undergoes diurnal cycles of accumulation during photoperiods and degradation during scotoperiods, providing a continuous carbon and energy supply for plant growth and development [2]. Under stress environmental conditions, plants typically activate starch remobilization to compensate for photosynthetic limitations, generating sugars and derived metabolites that function as reactive oxygen species (ROS) scavengers, osmolytes, and membrane/protein stabilizers [3]. Paradoxically, accumulating evidence demonstrates stress-induced starch hyperaccumulation, particularly under cold stress. Chilling-triggered starch deposition has been documented in diverse species including *Oryza sativa*, *Citrus junos*, and *Cucumis melo* [4–6]. Consistent with these observations, our prior investigations revealed that LNT stress induces chloroplast starch granule hypertrophy accompanied by photodamage exacerbation in tomato [7, 8]. The divergent starch metabolic responses across stress modalities remain mechanistically ambiguous. Critical knowledge gaps persist regarding the molecular regulation of amylase activities under stress conditions, particularly during nocturnal starch mobilization phases.

Photosynthetic carbon fixation through the Calvin-Benson cycle generates triose phosphates, which are subsequently converted into ADP-glucose via ADP-glucose pyrophosphorylase (AGPase). Transient starch granules are then deposited in chloroplasts as insoluble polyglucan reserves [9]. During the nocturnal phase, starch degradation commences with GWD/PWD-dependent phosphorylation of glucan chains, followed by hydrolytic cleavage by *β*-amylase (BAM) to yield maltose [10]. BAMs, belonging to the glycoside hydrolase family, are the principal enzymes driving starch catabolism in higher plants [11]. The *Arabidopsis thaliana* genome encodes nine BAM and BAM-like proteins [12], of which at least four (BAM1, BAM2, BAM3, and BAM4) are plastid-localized [13]. Among these, BAM1 and BAM3 exhibit the highest enzymatic activity and are considered the major functional isoforms [11]. Despite their structural similarity, BAM1 and BAM3 display distinct biochemical properties. BAM3 exhibits optimal activity under low temperatures and slightly acidic pH, making it crucial for nocturnal starch degradation in mesophyll cells [14]. BAM1, in contrast, functions more efficiently at higher temperatures and alkaline pH, and plays a specialized role in guard cells, particularly during early dawn [15]. Genetic evidence supports their non-redundant functions: *bam3* knockout mutants exhibit severe starch excess phenotypes due to impaired nighttime degradation [11], whereas *bam1* mutants primarily affect stomatal regulation rather than bulk leaf starch turnover [15].

BAM3 assumes a central role in chilling stress-induced starch catabolism, yet the precise mechanisms governing its enzymatic regulation remain elusive. Transcriptomic profiling has consistently demonstrated upregulation of *BAM3* under cold stress, implying potential enhancement of starch mobilization. Paradoxically, in planta enzymatic assays revealed a reduction in BAM3 catalytic activity following chilling treatment in *Arabidopsis thaliana* despite unaffected BAM1 activity [16]. This kinetic discrepancy was further corroborated in *bam1bam5* double mutants, where exclusive *BAM3* expression failed to prevent starch hyperaccumulation under cold stress [14]. Collectively, these findings suggest that chilling-induced starch retention arises from suppressed amylase activity rather than transcriptional repression. These seemingly conflicting results indicate potential post-translational regulation of BAM3 activity, particularly through glutathione-mediated inhibition as observed in C433 *in vitro* systems [16], though *in vivo* confirmation remains required.

The chloroplast vesiculation (CV) protein, encoded by nuclear genes, functions as a key regulator of chloroplast protein degradation under abiotic stress conditions, thereby modulating chloroplast homeostasis [17]. Stress- and age-induced chloroplast-targeted CV mediates the biogenesis of CV-containing vesicles (CCVs), which traffic inner envelope proteins, thylakoid components, and stromal polypeptides to the central vacuole or 26S proteasome system for degradation [17–19]. Overexpression of *CV* exacerbates stress susceptibility through attenuation of oxygen-evolving complex (OEC) activity and compromised photoprotective capacity [17, 18]. Beyond photosynthetic regulation, CV participates in carbon assimilation and source-sink regulation. Under water deficit conditions, CV physically interacts with chloroplast-localized glutamine synthetase (OsGS2), thereby modulating the coordination of carbon/nitrogen assimilation and photorespiratory flux [20]. Furthermore, the stress-responsive NAC transcription factor RD26 directly activates CV expression, orchestrating metabolic reprogramming in mitochondrial respiration and central carbon metabolism [21]. In tomatoes, *Slcv* silencing enhances source strength and sink capacity by optimizing photoassimilate allocation [22]. Notwithstanding these advances, the putative role of CV in starch metabolism remains underexplored, despite reduced starch accumulation observed in *CV*-silenced lines [23]. Emerging evidence highlights that starch metabolic perturbations compromise chloroplast functionality [24]. Our recent work uncovered a novel role for CV in chloroplast destabilization under low night temperature (LNT) stress [18], yet the mechanistic link to starch hyperaccumulation remains unresolved.

In this study, we found that *SlCV*-overexpressing (*SlCV*-OE) plants exhibited elevated leaf starch content and attenuated *β*-amylase activity under LNT stress, whereas *SlCV-*silenced lines displayed enhanced starch mobilization capacity. Strikingly, SlCV can directly interact with SlBAM3 and promote its degradation under LNT stress. The destruction of *SlBAM3* led to starch overaccumulation and greatly reduced the cold tolerance of tomato plants, whereas *SlBAM3* overexpression effectively complemented the LNT-sensitive phenotype in *SlCV*-OE lines. These findings provide unprecedented insight into cold-responsive starch metabolism in plants.

## Results

### Low temperature inhibits the degradation of starch at night

**Figure 1:**
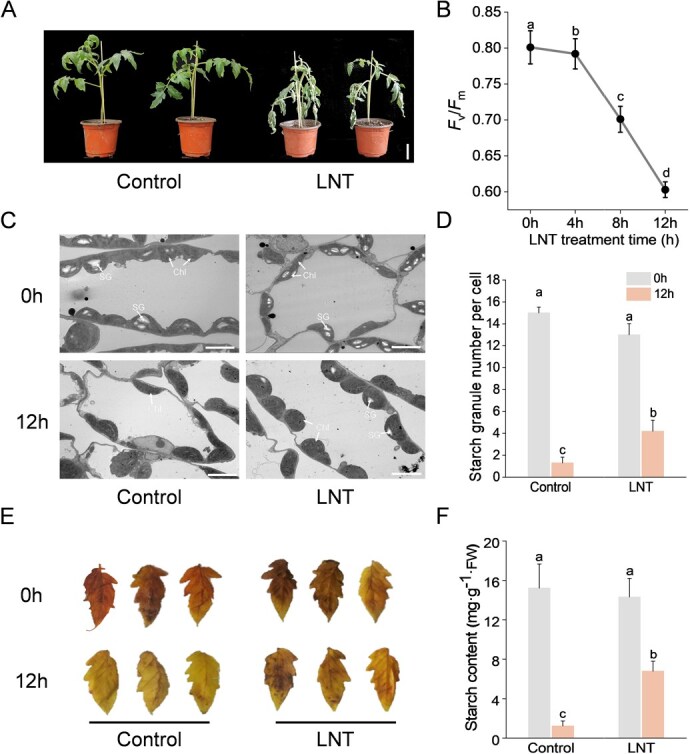
LNT inhibits the degradation of starch. A) Thephenotypes of tomato plants under LNT stress (bar, 5 cm). B) Changes in the maximum photochemical efficiency of PSII (*F*_v_/*F*_m_) at different time points of LNT treatment (one-way ANOVA). C) The ultrastructure of chloroplasts in tomato leaves before and after treatment. Chl., chloroplast; SG, starch granule. Bar, 5 μm. D) The starch granule number per cell (0 h, the end of day; 12 h, end of night; two-way ANOVA, treatment *P* > 0.05, time *P* < 0.001, interaction *P* < 0.001). E) I/KI staining analysis and starch content F) of tomato leaves (two-way ANOVA, treatment *P* < 0.01, time *P* < 0.001, interaction *P* < 0.001). At least three biological replicates were analyzed, with standard errors shown by vertical bars. Different letters indicate significant differences among treatments (*P* < 0.05).

To investigate the impact of low temperature on nighttime starch content changes in tomato leaves, we subjected tomato plants to low temperature treatment during the night. LNT stress caused visible wilting and significant decreased maximum quantum efficiency of photosystem II (*F*_v_*/F*_m_) relative to control plants (Fig. 1A and B). Ultrastructural analysis of chloroplasts revealed that LNT stress led to chloroplast expansion and deformation, causing a blurred thylakoid structure (Fig. 1C). In contrast to the degradation of chloroplast starch observed under normal temperature conditions, chloroplast starch granules in tomato leaves accumulated abundantly in the LNT environment (Fig. 1C and D). Additionally, tomato leaves exposed to LNT stress exhibited intensified iodine staining and higher starch content levels (Fig. 1E and F).

### Overexpression of *SlCV* exacerbates starch accumulation under LNT stress

**Figure 2:**
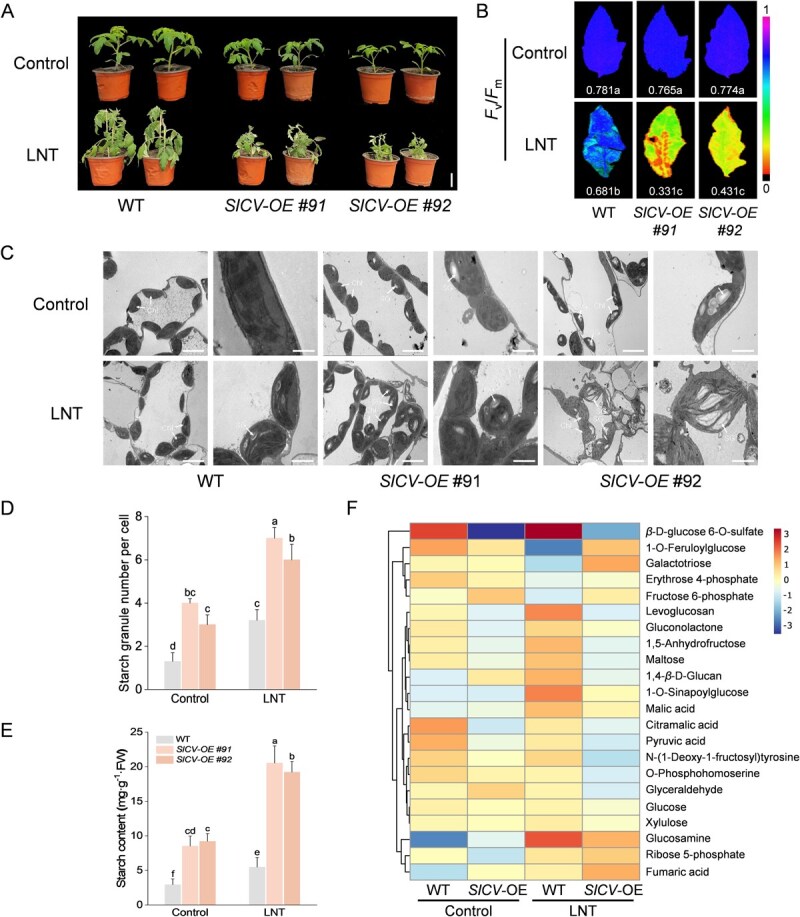
*SlCV-*OE exacerbates starch accumulation under LNT stress. A) The phenotypes of WT and *SlCV*-OE plants before and after LNT treatment (bar, 5 cm). B) The maximum photochemical efficiency of PSII (*F*_v_/*F*_m_) of WT and *SlCV*-OE plants before and after LNT treatment. C)The ultrastructure of chloroplasts in tomato leaves before and after treatment. Chl., chloroplast; SG, starch granule. Bar, 5 μm or 500 μm. D) The starch granule number per cell (two-way ANOVA, treatment *P* < 0.001, genotype *P*< 0.001, interaction *P* < 0.05). E) Starch content of tomato leaves (two-way ANOVA, treatment *P* < 0.001, genotype *P* < 0.001, interaction *P* < 0.001). F) The content changes of starch and sugar metabolism-related metabolites in WT and *SlCV*-OE plants detected by non-targeted metabolomics under control and LNT stress conditions. At least three biological replicates were analyzed, with standard errors shown by vertical bars. Different letters indicate significant differences among treatments (*P* < 0.05).

CV plays a crucial role in regulating chloroplast structure and stress resistance, and can be rapidly induced by LNT (Supplementary Fig. S1A, [18]). We generated *SlCV* overexpression plants (*SlCV*-OE #91, *SlCV*-OE #92) to explore the association between SlCV and starch metabolism in tomato leaves under LNT stress (Supplementary Fig. S1B, [18, 25]). Remarkably, *SlCV*-overexpressing line plants exhibited substantially shorter stature than wild-type (WT) controls (Supplementary Fig. S1C and D). Subsequent LNT treatment revealed that *SlCV*-overexpression led to a heightened sensitivity phenotype, characterized by increased wilting (Fig. 2A), decreased *F*_v_/*F*_m_ values (Fig. 2B), disassembly of photosystem complexes, and disorganized chloroplast structure (Supplementary Fig. S1E). Notably, analysis of chloroplast ultrastructure demonstrated an elevated presence of starch grains in *SlCV*-overexpressing tomato leaves, even at ambient temperature (Fig. 2C and D). This observation was further supported by potassium iodide staining and starch content analysis, confirming that tomato leaves with *SlCV*-overexpression accumulated more starch under LNT stress (Fig. 2E; Supplementary Fig. S1F).

Subsequently, we conducted a metabolomic analysis of *SlCV*-overexpression and WT plant leaves exposed to room temperature and LNT to elucidate the involvement of SlCV in starch metabolism. Metabolomic profiling showed *SlCV*-overexpression altered metabolite levels under both normal and LNT conditions, with 757 up-regulated and 1033 down-regulated metabolites at room temperature (Supplementary Fig. S2A), compared to 2254 up-regulated and 1749 down-regulated under LNT stress (Supplementary Fig. S2B). Kyoto Encyclopedia of Genes and Genomes (KEGG) analysis revealed consistent enrichment in carbon metabolism pathways across all conditions (Supplementary Fig. S2C). Notably, in plants overexpressing *SlCV*, the majority of metabolites associated with starch and glucose metabolism were down-regulated under LNT stress, particularly maltose, a product of starch degradation, thus providing further evidence of SlCV’s ability to suppress starch degradation during the night.

### *SlCV* silencing promotes starch degradation under LNT stress

**Figure 3:**
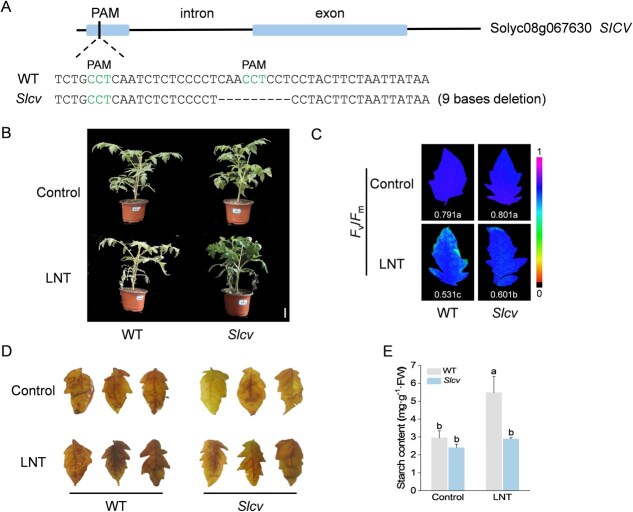
SlCV silencing promotes starch degradation under LNT stress. A) *Slcv* mutant identification. B) The phenotypes of WT and *Slcv* mutant plants before and after LNT treatment (bar, 5 cm). C) The maximum photochemical efficiency of PSII (*F*_v_/*F*_m_) of WT and *Slcv* mutant plants before and after LNT treatment. D) I/KI staining analysis and starch content E) of tomato leaves (two-way ANOVA, treatment *P* < 0.001, genotype *P* < 0.001, interaction *P* < 0.001). At least three biological replicates were analyzed, with standard errors shown by vertical bars. Different letters indicate significant differences among treatments (*P* < 0.05).

We generated *Slcv* mutant tomato plants by deleting 9 bases in the gene and assessed their photosynthetic performance and starch levels under LNT stress (Fig. 3A). As expected, the *Slcv* mutants exhibited improved plant morphology and higher *F*_v_/*F*_m_ values following exposure to LNT (Fig. 3B and C). Potassium iodide staining revealed lighter leaf coloration in *Slcv* mutant plants compared to WT plants under LNT stress (Fig. 3D). Additionally, a marked reduction in starch content was observed in the *Slcv* mutants relative to WT plants under LNT stress (Fig. 3E).

### SlCV induced transcriptional reprogramming of starch metabolism under LNT stress

**Figure 4:**
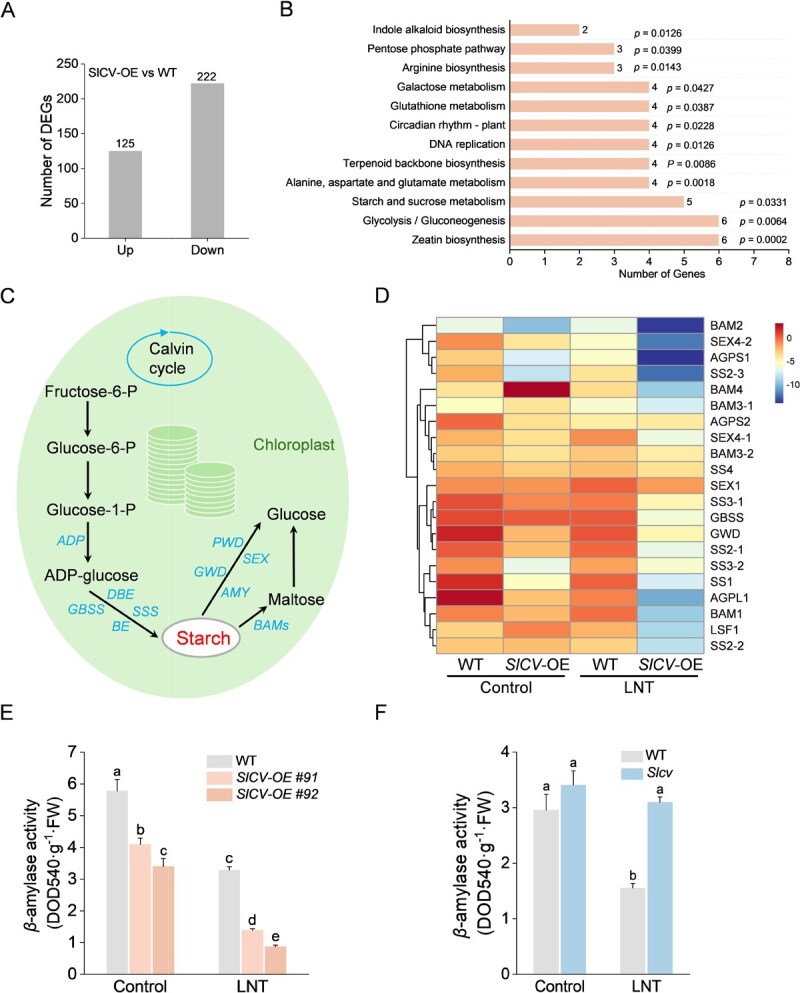
RNA-seq analysis of *SlCV*-OE plants. A) The Number of differentially expressed genes (DEGs) between *SlCV*-OE and WT plants under the control condition. B) Kyoto Encyclopedia of Genes and Genomes (KEGG) enrichment analysis of differentially expressed genes (DEGs) in WT and *SlCV*-OE plants. C) Schematic diagram of starch synthesis and degradation. Key genes are indicated in italics. D) qRT-PCR analysis of the starch metabolism key genes’ transcriptional levels in WT and *SlCV*-OE plants under control and LNT stress conditions. E) The *β*-amylase activity of WT and *SlCV*-OE plants under control and LNT stress conditions (two-way ANOVA, treatment *P* < 0.001, genotype *P* < 0.001, interaction *P* > 0.05). F) The *β*-amylase activity of WT and *Slcv* mutant plants under control and LNT stress conditions (two-way ANOVA, treatment *P* < 0.001, genotype *P* < 0.001, interaction *P* < 0.001). At least three biological replicates were analyzed, with standard errors shown by vertical bars. Different letters indicate significant differences among treatments (*P* < 0.05).

The accumulation of starch may result from enhanced synthesis and restricted degradation. To elucidate the precise mechanism of SlCV in starch metabolism, we conducted RNA sequencing to analyze the global transcriptomic profiles of *SlCV*-overexpressing and WT plants, specifically examining starch-related differentially expressed genes (DEGs). Principal component analysis (PCA) demonstrated a strong correlation between *SlCV*-OE and WT groups (Supplementary Fig. S3A). Comparative transcriptomics identified 347 DEGs in *SlCV*-OE and WT plants, with 222 genes showing down-regulation (Fig. 4A; Supplementary Fig. S3B). Gene Ontology (GO) enrichment analysis revealed significant enrichment of DEGs in terms related to stress response and chloroplast thylakoids (Supplementary Fig. S3C). Notably, KEGG enrichment analysis indicated a significant enrichment of DEGs in pathways related to starch and sucrose metabolism (Fig. 4B), highlighting the association between SlCV and carbon fixation processes, such as starch and sugar metabolism. Starch synthesis commences with fructose and glucose generated by the Calvin cycle and culminates in its degradation by amylase into maltose and glucose, which are then transported out of the chloroplasts [10]. Various genes, such as *ADP*, *SSS*, and *GBSS* participate in starch synthesis, while *BAMs*, *SEX*, *GWD*, and *LSF* genes regulate starch degradation (Fig. 4C). Through RNA-seq analysis, we assessed the expression levels of starch metabolism-related genes under control and LNT stress using qRT-PCR. Compared to WT plants, overexpression of *SlCV* resulted in the downregulation of most starch synthesis and degradation genes under both control and LNT conditions. Remarkably, genes associated with starch degradation, notably BAMs (Fig. 4D). Given that BAMs predominantly encode *β*-amylase, we investigated *β*-amylase activity in various plants. Overexpression of *SlCV* significantly inhibited *β*-amylase activity in tomato leaves under both control and LNT stress conditions (Fig. 4E). Conversely, *β*-amylase activity was significantly increased in *Slcv* mutant plants under LNT stress (Fig. 4F), suggesting that the starch accumulation triggered by SlCV is linked to reduced *β*-amylase activity.

### SlCV interacts with SlBAM3 and promotes its degradation

**Figure 5:**
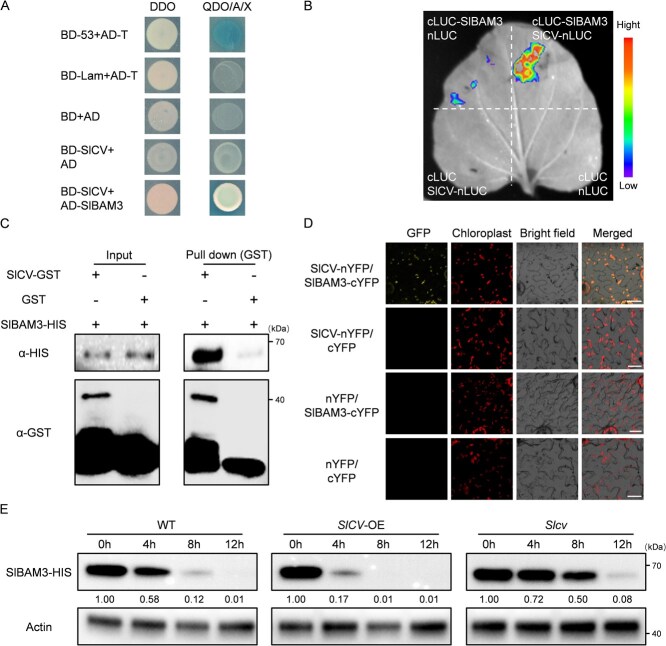
SlCV interacts with SlBAM3 and promotes its degradation. A) Yeast two-hybrid experiments verified that SlCV and SlBAM3 interacted. DDO, SD medium lacking Trp and Leu; QDO, SD medium lacking Trp, Leu, His, and Ade; X, TDO medium containing x-α-gal. A. The empty plasmids were used as controls. The AD-T and P53 were used as the positive control. AD-T and Lam were used as the negative control. B) Luciferase complementation imaging assay showing that SlCV interacts with SlBAM3 in *Nicotiana benthamiana* leaves. *Agrobacterium tumefaciens* strain EHA105 harboring different constructs was infiltrated into *Nicotiana benthamiana* leaves and examined after 72 h. Similar results were obtained in three independent experiments. C) *In vitro* pull-down investigation demonstrating the interaction of SlCV with SlBAM3. Recombinant GST-tagged SlCV protein (SlCV-GST) was linked to glutathione sepharose beads, subjected to incubation with recombinant HIS-tagged SlBAM3 protein (SlBAM3-HIS), and immunoblotted using anti-GST and anti-HIS antibodies. D) Interaction of SlCV and SlBAM3 detected by bimolecular fluorescence complementation assay analysis. SlBAM3 was fused to the C-terminal fragment of YFP (cYFP), and SlCV was fused to the N-terminal fragment of YFP (nYFP). The construct combinations were co-transformed into *Nicotiana benthamiana* leaves and expressed for 48 h. The signal was detected by confocal microscopy (bar, 25 μm). E) Cell-free degradation assays. The SlBAM3-HIS recombinant protein purified from *Escherichia coli* was co-incubated with the total protein extracts from leaves of WT, *SlCV*-OE, and *Slcv* plants treated with LNT stress. Immunoblot analysis was performed using anti-HIS antibody at different time points. The anti-Actin served as a loading control

In previous research, CV was primarily implicated in the cytosol degradation of chloroplast proteins [18]. We hypothesize that CV may also influence the stability of proteins associated with starch metabolism. To investigate protein interactions, we performed yeast two-hybrid (Y2H) assays. SlBAM1, SlBAM2, SlBAM3, SlSEX4, SlSS1, SlADP, and SlCV were chosen for Y2H analysis. Among all tested combinations, only BD-SlCV+AD-SlBAM3 and BD-SlCV+AD-SlSEX4 exhibited growth on SD-QDO medium (Fig. 5A; Supplementary Fig. S4A), indicating the possibility of specific protein interactions between SlCV and starch-degrading enzymes. Given the different variations of *β*-amylase activity in SlCV-related plants and the fact that BAM3 is considered the main gene controlling starch nighttime degradation [11], we focused on exploring the relationship between SlCV and SlBAM3. The firefly luciferase (LUC) complementation imaging (LCI) assay further confirmed their interaction *in vivo*. A strong fluorescence signal was detected when co-expressing the SlCV construct (SlCV-nLUC) fused to the N-terminus of LUC and the recombinant plasmid of SlBAM3 connected to the C-terminus of LUC (cLUC-SlBAM3) in *Nicotiana benthamiana* leaves (Fig. 5B). *In vitro*, we obtained the recombinant proteins of SlCV and SlBAM3 with GST tag and HIS tag, respectively, and performed GST pull-down assay. SlBAM3-HIS was detected in the enriched components of SlCV-GST but not in the negative control (Fig. 5C). Next, when the recombinant plasmids of SlCV-GFP and SlBAM3-GFP driven by CaMV35S promoter were expressed in *Nicotiana benthamiana* leaves, we observed green fluorescence signals coinciding with the autofluorescence of chloroplasts under a confocal laser scanning microscope, indicating that SlCV and SlBAM3 were located within the chloroplasts (Supplementary Fig. S5). Consistent with these findings, chloroplast-localized YFP fluorescence was detected in *Nicotiana benthamiana* leaves co-expressing SlCV-nYFP and SlBAM3-cYFP fusion constructs, while control infiltrations showed no detectable signal (Fig. 5D).

**Figure 6:**
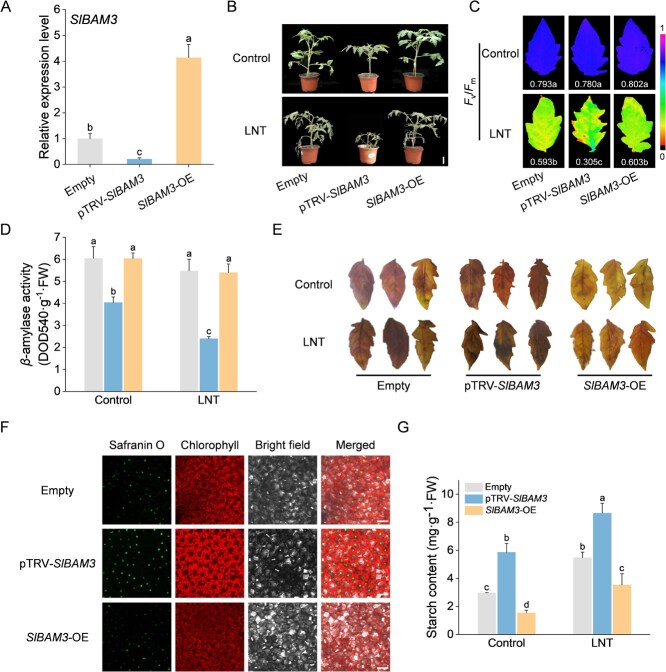
SlBAM3 enhances the LNT tolerance of tomato plants. A) Relative expression of SlBAM3 in *SlBAM3-*silenced (pTRV-*SlBAM3*) and *SlBAM3-*transient overexpression (*SlBAM3*-OE) plants (one-way ANOVA). B) The phenotypes of WT (Empty), pTRV-*SlBAM3,* and *SlBAM3*-OE plants before and after LNT treatment (bar, 5 cm). C) The maximum photochemical efficiency of PSII (*F*_v_/*F*_m_) of WT (Empty), pTRV-*SlBAM3,* and *SlBAM3*-OE plants before and after LNT treatment. D) The *β*-amylase activity of tomato leaves under control and LNT stress conditions (two-way ANOVA, treatment *P* < 0.001, genotype *P* < 0.001, interaction *P* < 0.01). E) I/KI staining analysis of tomato leaves. F) Starch granules in mesophyll cells. Observation of starch granules’ fluorescence (stained with safranin O) in the WT (empty), pTRV-*SlBAM3,* and *SlBAM3*-OE plants after LNT stress (bar, 25 μm). G) Starch content of tomato leaves (two-way ANOVA, treatment *P* < 0.001, genotype *P* < 0.001, interaction *P* > 0.05). At least three biological replicates were analyzed, with standard errors shown by vertical bars. Different letters indicate significant differences among treatments (*P* < 0.05).

**Figure 7:**
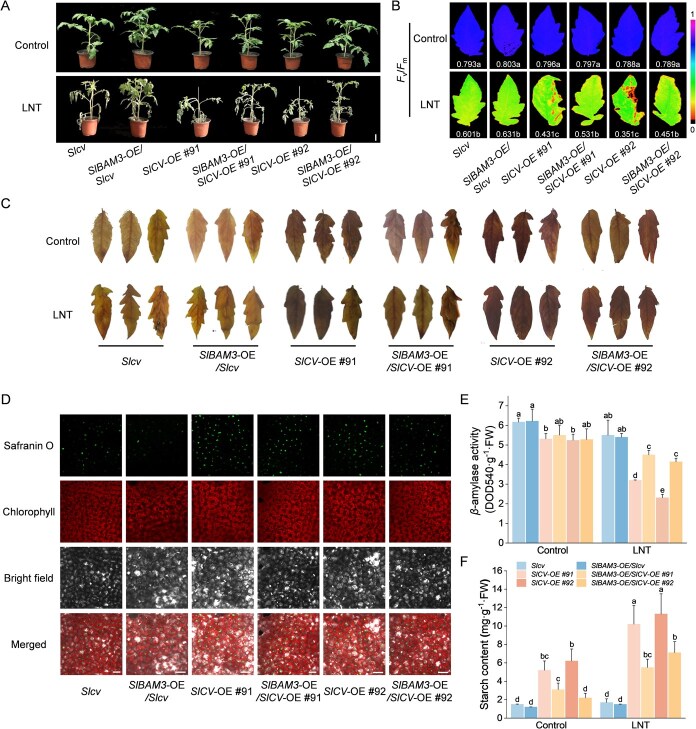
SlBAM3 is required for SlCV-mediated starch accumulation under LNT. A) The phenotypes of *SlBAM3-*transient overexpression in *Slcv* mutant (*SlBAM3*-OE/*Slcv*) or SlCV-OE lines (*SlBAM3*-OE/*SlCV-*OE #91, *SlBAM3*-OE/*SlCV-*OE #92) plants before and after LNT treatment (bar, 5 cm). C) The maximum photochemical efficiency of PSII (*F*_v_/*F*_m_) of *SlBAM3-*transient overexpression in *Slcv* mutant (*SlBAM3*-OE/*Slcv*) or SlCV-OE lines (*SlBAM3*-OE/*SlCV-*OE #91, *SlBAM3*-OE/*SlCV-*OE #92) plants before and after LNT treatment. C) I/KI staining analysis of tomato leaves. D) Starch granules in mesophyll cells. Observation of starch granules’ fluorescence (stained with safranin O) in the tomato leaves after LNT stress (bar, 25 μm). E) The *β*-amylase activity of tomato leaves (two-way ANOVA, treatment *P* < 0.001, genotype *P* < 0.001, interaction *P* < 0.001). F) The starch content of tomato leaves (two-way ANOVA, treatment *P* < 0.001, genotype *P* < 0.001, interaction *P* < 0.001). At least three biological replicates were analyzed, with standard errors shown by vertical bars. Different letters indicate significant differences among treatments (*P* < 0.05).

The results confirmed the physical interaction between SlCV and SlBAM3 while also raising new questions regarding whether SlCV mediates the degradation of SlBAM3. To investigate this, we conducted a cell-free proteasome degradation assay to assess the stability of SlBAM3 across different plant lines. Total leaf proteins from WT, *SlCV*-OE, and *Slcv* mutant plants under LNT stress were extracted and incubated with *in vitro* purified SlBAM3-HIS. Reactions were terminated at 0, 4, 8, and 12 hours, and Western blot analysis using HIS antibody was performed to quantify the protein levels of SlBAM3. As illustrated in Fig. 5E and Supplementary Fig. S4B, compared with WT plants, overexpression of *SlCV* significantly accelerated the degradation rate of SlBAM3, with lower levels of SlBAM3 protein being observed after just 4 hours of incubation. In contrast, *SlCV*-silencing significantly delayed the degradation of SlBAM3. These findings suggest that SlCV interacts with SlBAM3 in chloroplasts and facilitates the degradation of SlBAM3 under LNT stress.

### SlBAM3 enhances the LNT tolerance of tomato plants

To investigate the impact of SlBAM3 on tomato response to LNT stress, we developed experimental materials for both silencing and overexpressing *SlBAM3* in tomato plants. Transcriptional analysis confirmed successful downregulation and upregulation of SlBAM3 in *SlBAM3*-silenced plants (pTRV-*SlBAM3*) and *SlBAM3*-overexpressing plants (*SlBAM3*-OE), respectively (Fig. 6A). In contrast to control and *SlBAM3*-OE plants, *SlBAM3*-silenced plants displayed reduced plant height and more pronounced wilting and necrosis symptoms under LNT stress conditions (Fig. 6B; Supplementary Fig. S6A). Additionally, *F*_v_/*F*_m_ was significantly decreased in *SlBAM3*-silenced plants under LNT stress, indicating heightened photosynthetic damage (Fig. 6C). Consistent with the essential role of BAM3 in *β*-amylase activity, we observed significantly reduced *β*-amylase activity in *SlBAM3*-silenced plants compared to controls (Fig. 6D), confirming the successful silencing of SlBAM3. To further evaluate the impact of SlBAM3 on starch metabolism, we analyzed starch accumulation in various plant materials under both stress and nonstress conditions. Potassium iodide staining revealed darker coloration in the leaves of *SlBAM3*-silenced plants, indicative of higher starch accumulation, whereas *SlBAM3*-OE plants exhibited lighter staining (Fig. 6E). Additionally, we employed Safranin O staining to visualize starch granules in leaves under LNT stress using laser confocal microscopy. *SlBAM3*-silenced plants displayed more intense green fluorescence signals compared to control and *SlBAM3*-OE plants, further supporting the accumulation of starch in the silenced lines (Fig. 6F). Quantitative analysis of starch content further confirmed that *SlBAM3* silencing led to increased starch accumulation under both room temperature and LNT conditions, while *SlBAM3* overexpression enhanced starch degradation (Fig. 6G).

### SlBAM3 is required for SlCV-mediated starch accumulation under LNT

Our study confirmed the role of SlCV in regulating the stability of the SlBAM3 protein. To further investigate whether the increased SlBAM3 could alleviate the sensitivity of SlCV to LNT stress and starch metabolism, we overexpressed *SlBAM3* driven by the CaMV35S promoter in *Slcv* mutants and *SlCV*-OE plants (*SlCV*-OE #91, *SlCV*-OE #92, Supplementary Fig. S6B). *SlBAM3* overexpression in *SlCV-*OE lines enhanced LNT stress tolerance, evidenced by improved plant morphology and higher *F*_v_*/F*_m_ values under LNT conditions. Notably, *SlBAM3*-OE plants with the *Slcv* mutant background partially restored the LNT stress tolerance of *Slcv* mutants (Fig. 7A and B). As expected, *SlBAM3* overexpression alleviated the dark staining of leaves by potassium iodide and reduced the green fluorescence signals of starch granules labeled by Safranin O in both *Slcv* mutants and *SlCV*-OE plants (Fig. 7C and D). Furthermore, measurements of starch content and *β*-amylase activity revealed that *SlBAM3* overexpression significantly enhanced *β*-amylase activity in *SlCV*-OE plants under LNT stress, thereby markedly reducing starch accumulation (Fig. 7E and F). To further validate the role of SlBAM3, we silenced *SlBAM3* in *Slcv* mutants and *SlCV*-OE plants (Supplementary Fig. S6C). Similar to the effects observed in pTRV-*SlBAM3* plants alone, the downregulation of *SlBAM3* reduced plant height and increased sensitivity to LNT stress in both *Slcv* mutants and *SlCV-*OE plants (Supplementary Fig. S6D). Additionally, *SlBAM3*-silencing significantly decreased *β*-amylase activity, leading to further starch accumulation in the background plants, particularly in *SlCV*-OE plants (Supplementary Fig. S6E and F). These results establish that SlBAM3 is a crucial downstream effector of SlCV in regulating starch accumulation.

## Discussion

**Figure 8:**
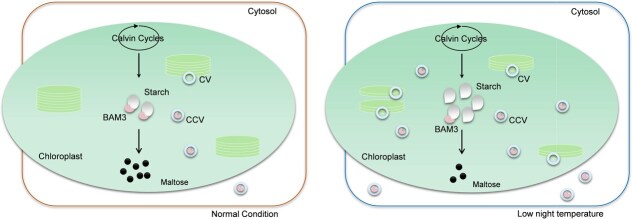
A proposed model that SlCV affects starch metabolism through regulating SlBAM3 stability under low night temperature stress. Compared to normal conditions, low night temperature induces more SlCV, which targets the chloroplast and interacts with SlBAM3, participating in the degradation of SlBAM3 outside the chloroplast by forming SlCV/SlBAM3-containing vesicles (CCV). The decreased levels of SlBAM3 hinder the timely degradation of starch, ultimately resulting in starch accumulation within the chloroplast and a reduction in maltose levels during low night temperature stress.

Precise regulation of energy allocation is critical for optimal plant growth and development, with modulation occurring in response to developmental cues and environmental perturbations. Leaf transient starch content typically decreases under stress conditions, including salinity and drought ([26, 27]). The efficient breakdown of starch helps maintain metabolite levels in the Calvin-Benson cycle and facilitates the redistribution of carbon for the production of osmoprotectants or cryoprotectants. Notably, under specific experimental conditions, particularly during extended or intense stress exposure or nocturnal stress periods, plants exhibited significant starch accumulation [28, 29]. In our previous and recent investigations, LNT stress promoted starch accumulation and hindered the redistribution of photosynthetic products. As a result, a greater number of starch granules were retained in the chloroplasts of tomato leaves at dawn (Fig. 1; [7, 8]). This metabolic perturbation negatively impacts photosynthetic carbon remobilization and the production of stress-protective compounds, ultimately compromising photosynthetic performance and stress adaptation capacity.

Stress and darkness induced CV targets chloroplasts and leads to the degradation of thylakoid proteins, stromal proteins, and even entire chloroplasts [17]. Under LNT stress, SlCV participates in the light-response mechanisms of tomato leaves via modulating the non-photochemical quenching (NPQ) process mediated by SlPsbS [18]. In this study, we further demonstrate that *SlCV* overexpression (*SlCV*-OE) disrupts chloroplast ultrastructure under LNT stress, causing thylakoid membrane disassembly and severe photodamage (Fig. 2). Beyond its role in chloroplast protein stability, CV also influences photosynthetic carbon metabolism. Under water stress and elevated CO_2_, *OsCV*-silenced rice plants exhibit enhanced carbon and nitrogen assimilation rates and photorespiration [20, 30]. In Barros et al. [23] study, the sugar and starch content in Arabidopsis leaves declined rapidly upon *CV* silencing. Consistently, under LNT stress, we observed excessive starch accumulation in *SlCV*-OE plants, whereas *Slcv* mutants exhibited efficient starch degradation (Figs 2 and 3). These findings suggest that starch accumulation may contribute to the stress-sensitive phenotype of *CV*-overexpressing plants, rather than being solely a consequence of chloroplast structural damage. In addition, starch overaccumulation itself may exacerbate chloroplast degradation, as previously reported in Arabidopsis [31]. Notably, starch, a storage polysaccharide, serves distinct physiological roles. Transitory starch fuels nocturnal metabolism, whereas long-term starch reserves support vegetative growth, reproduction, or stress adaptation. Preventing starch metabolism can affect plant growth [31], potentially explaining the growth retardation observed in Sl*CV*-OE plants (Fig. 2; Supplementary Fig. S1C), as *SlCV*-OE lines also exhibit elevated starch levels even under normal growth conditions (Fig. 2).

Starch metabolism is tightly regulated through the coordinated action of enzymes governing its synthesis and degradation [9]. The process initiates with ADP-glucose synthesis, and polymerization into starch is mediated by starch synthases (SSs), starch branching enzymes (SBEs), and debranching enzymes (DBEs) [9, 32]. Before degradation, starch phosphorylation disrupts granule structure, enabling enzymatic breakdown by *β*-amylase (BAM) and *α*-amylase into transportable sugars for cytosolic metabolism [3, 33]. In the present experiment, multiomics analyses uncovered distinct transcriptional and metabolic reprogramming associated with SlCV-mediated starch metabolism regulation. Under both control and LNT conditions, differential metabolites were significantly enriched in carbon metabolism pathways (Supplementary Fig. S2C and D), underscoring the pivotal role of SlCV in modulating photosynthetic carbon allocation. RNA-Seq and RT-qPCR revealed widespread downregulation of starch metabolic genes in *SlCV*-OE plants under LNT stress, particularly those encoding amylases (Fig. 4D). Consistent with this, *SlCV*-OE plants exhibited reduced amylase activity, whereas the opposite trend was observed in *SlCV*-silenced plants (Fig. 4E and F). The levels of starch degradation products, maltose and glucose, also decreased significantly in *SlCV*-OE plants (Fig. 2F). This finding suggests that starch accumulation in these plants results primarily from impaired degradation rather than enhanced synthesis, given that the substrates for starch synthesis also decreased in *SlCV*-OE plants (Fig. 2F).

The modulation of protein stability by CV frequently entails interactions with specific proteins. Our previous work demonstrated CV’s interactions with both the thylakoid protein PsbS and the peroxisomal protein CAT3, thereby coordinating photoprotective responses and ROS homeostasis under stress conditions [18, 25]. To understand the mechanism of CV in starch metabolism, we systematically examined its potential interactions with starch metabolic enzymes. Intriguingly, CV seems to interact specifically with starch-degrading enzymes rather than starch synthases (Supplementary Fig. S4), further accentuating the connection between CV and starch degradation pathways. Through comprehensive *in vitro* and *in vivo* analyses, we established that CV physically interacts with BAM3 and, more significantly, promotes BAM3 protein turnover under LNT stress (Fig. 5). This represents a novel post-translational regulatory mechanism controlling BAM3 abundance. Within the BAM enzyme family, BAM3 has been identified as the predominant isoform mediating nighttime starch breakdown in leaf mesophyll tissues, and it exhibits the capability to hydrolyze leaf starch completely [3, 34]. The physiological significance of BAM3 regulation is underscored by its stress-responsive activation. Cold stress triggers rapid BAM3 induction, and genetic evidence links BAM3 activity to stress adaptation [35]. *bam3* mutants display enhanced drought sensitivity and impaired photosynthesis, whereas *BAM3* overexpression confers drought tolerance (Zhang et al. 2023). Our observations on tomato corroborate these findings. *SlBAM3*-silenced lines exhibited compromised stress resistance concomitant with starch overaccumulation (Fig. 6). Remarkably, genetic complementation experiments demonstrated that *SlBAM3* overexpression in *SlCV*-OE plants not only reduced starch accumulation but also ameliorated the LNT-sensitive phenotype (Fig. 7). Conversely, concurrent silencing of *SlBAM3* in *SlCV*-OE plants exacerbated stress damage and further elevated starch levels under LNT (Supplementary Fig. S6). These genetic interactions establish that SlCV-mediated stress sensitivity and starch accumulation operate partially through SlBAM3 regulation.

This study elucidates the molecular mechanisms underlying SlCV-mediated starch accumulation under LNT stress, revealing multilayered regulation at both transcriptional and metabolic levels in starch anabolic and catabolic pathways. The study focuses on elucidating the regulatory cascade* through which SlCV promotes the degradation of the SlBAM3 protein under LNT conditions, thereby affecting *β*-amylase activity and ultimately promoting starch accumulation (Fig. 8). Our results characterize a previously unrecognized mechanism of SlBAM3 protein degradation under LNT stress, establishing SlCV as a key regulator of starch catabolism. Demonstrating SlCV orchestrates comprehensive regulation of photosynthesis, spanning from primary carbon metabolism to photoprotective mechanisms.

## Materials and methods

### Plant material and growth conditions

The ‘Ailsa Craig’ cultivar of tomato (*Solanum lycopersicum*) served as the wild type (WT) in this research. Our lab was tasked with preserving the seeds for both *SlCV*-OE and *Slcv* [18]. As done previously, we employed the virus-induced gene silencing (VIGS) method utilizing tobacco rattle virus (TRV)-based vectors (pTRV1/2) to downregulate the *SlBAM3* genes ([25, 36]). The complete *SlBAM3* sequence was cloned into the plant binary vector PRI101-AN and transiently overexpressed in tomato leaves through *Agrobacterium tumefaciens* infiltration. The effectiveness of silencing and overexpression of *SlBAM3* was evaluated using RT-qPCR.

Tomato plants were grown under controlled environmental conditions (28°C day/18°C night, 60% RH, 600 μmol m^−2^ s^−1^ PPFD, 12 h photoperiod) until the five-leaf stage. For cold stress experiments, plants were exposed to 4°C for 12 h (nocturnal period), with 18°C maintained as the control condition. Harvest the samples at the end of treatment. All physiological measurements were performed on the fourth fully expanded true leaf. For molecular analyses, treated leaves were either processed fresh or flash-frozen in liquid nitrogen for −80°C storage. Each experimental group included five biological replicates.

### Starch iodine test

Fresh leaves were harvested and decolorized in 95% ethanol, then immersed in an iodine/potassium iodide (5%/10%) solution. After the leaves were stained, the samples were observed and photographed with a camera.

### Starch content and *β*-amylase activity assay

Leaf starch levels and *β*-amylase activity were measured using the Starch Content Assay Kit and the *β*-amylase Activity Assay Kit (Solarbio, China), respectively. The experiments were conducted under the manufacturer’s instructions.

### Safranin O staining

According to earlier findings [37], small sections of tender tomato leaves were sliced and immersed in a safranin O solution (5 mg/ml, Sigma, Germany) for a duration of 0.5 to 1 h. This step facilitated the penetration of the dye through the stomata and cut into the tissue. Afterward, any excess dye was washed away using PBS buffer, and the samples were examined with a confocal laser scanning microscope (LSM880NLO, Zeiss, Germany). Using spectral unmixing, stained starch granules were clearly distinguished from chloroplast autofluorescence, demonstrating excellent contrast.

### The maximum quantum yield of PSII (*F*_v_/*F*_m_)

*F*_v_/*F*_m_ values were obtained using the MAXI-Imaging-PAM with blue LEDs and the imaging fluorometer software Win (version 2.46i, Heinz Walz, Effeltrich, Germany), as outlined in prior research [38]. After 30 min dark adaptation, measurements were initiated with a 300 ms saturation pulse (10 000 μmol m^−2^ s^−1^) to determine *F*_m_, while *F*_o_ was recorded under weak measuring light (<0.1 μmol m^−2^ s^−1^). The parameter was calculated as (*F*_m_ − *F*_o_)/*F*_m_.

### Blue-native PAGE

Collect the LNT-treated leaves and thoroughly grind them in a pre-cooled STN buffer (0.4 M Sucrose, 50 mM Tris pH = 7.6, 10 mM NaCl). Thylakoid membranes were isolated as described by Dong et al. [39]. Adjust the thylakoid membrane solution concentration for each treatment to 1 mg ml^−1^, and perform electrophoresis using a gradient gel of Native PAGE™ (Invitrogen, USA) at 4°C. After electrophoresis, stain the gel with a FastBlue protein rapid staining solution (Biosharp, China). After destaining, take photographs and save them.

### Chloroplast ultrastructure

Randomly select the fourth fully unfolded leaf from the top of the plant and perform electron microscopy examination after LNT treatment. Leaf sections were prepared and examined using transmission electron microscopy (Hitachi H7650, Japan, 75 kV) following established protocols [40].

### RNA extraction and expression analysis

RNA was extracted in total, followed by the synthesis of cDNA and the execution of RT-qPCR, as previously detailed [38]. All PCR primer sequences are provided in Supplementary Table S1. The internal reference gene used in this study was *SlActin*.

### Transcriptome and metabolic profiling

The transcriptomic analysis and non-targeted metabolomics were carried out by LC Bio Technology Co., Ltd. Total RNA was extracted using TRIzol reagent (Invitrogen Company, Waltham, MA, USA), with subsequent library preparation using the NEBNext Ultra RNA kit (New England Biolabs Company, USA). High-throughput sequencing was conducted on an Illumina Novaseq 6000 platform (LC Bio Technology Co., Ltd., China). Gene expression quantification employed FPKM normalization, followed by bioinformatics analysis to identify differentially expressed genes (DEGs) (*P* < 0.05) and functional annotation (Supplementary Table S3). Three biological replicates were analyzed per experimental group.

To conduct Non-targeted Metabolomics, we adapted the approach described by Turner et al. [41]. For metabolite profiling, fresh samples were lyophilized and homogenized before extraction with 70% methanol (1 ml per 100 mg sample). After centrifugation (10 000 × g, 10 min, 4°C), the supernatant was filtered (0.22 μm) and analyzed by UPLC-MS/MS (Shimadzu UFLC CBM30A coupled with AB Sciex 4500 QTRAP). Identified differential metabolites are documented in Supplementary Table S4.

### Yeast two-hybrid assays

To investigate protein–protein interactions, the full-length *SlCV* and *SlBAM3* coding sequences were cloned into pGBKT7 (bait) and pGADT7 (prey) vectors, respectively. The recombinant plasmids were co-transformed into Yeast two-hybrid (Y2H) Gold Yeast cells (*Saccharomyces cerevisiae*) and initially selected on DDO (−Leu/−Trp) medium. Putative interactors were verified on QDO plates containing X-α-gal (40 μg/ml) and AbA (150 ng/ml). Control experiments included BD-53/AD-T (positive) and BD-Lam/AD-T (negative) combinations. All plates were incubated at 30°C for 3–5 days.

### Luciferase complementation imaging assays

*SlCV* and *SlBAM3* coding sequences were directionally cloned into pCAMBIA1300 vectors containing N-terminal (nLUC) or C-terminal (cLUC) luciferase fragments. The recombinant plasmids were co-delivered into *Nicotiana benthamiana* leaves through *Agrobacterium tumefaciens*-mediated transformation. After 72 h postinfiltration, luminescent signals were detected following application of 0.2 mM luciferin substrate using a cooled CCD imaging system (Berthold Technologies, Germany).

### Subcellular localizationwere

*SlCV* and *SlBAM3* coding sequences (stop codon removed) were cloned into pCAMBIA1300-GFP and transiently expressed in *Nicotiana benthamiana* via *Agrobacterium tumefaciens* (GV3101) infiltration. Confocal microscopy (Zeiss LSM880) performed 48 h post-transformation revealed GFP fluorescence (excitation 488 nm/emission 520–540 nm) with chloroplast autofluorescence (680–750 nm), confirming plastid targeting.

### Bimolecular fluorescence complementation assay

The *SlCV* and *SlBAM3* were directionally cloned into pSPYNE/pSPYCE vectors (35S promoter) to create YFP fusion constructs. *Agrobacterium tumefaciens* GV3101 strains harboring different plasmid combinations were grown overnight, then incubated for 2 h before being infiltrated into *Nicotiana benthamiana* plants. Reconstituted YFP signals were analyzed by confocal microscopy (Zeiss LSM880) after 48 h, using standard YFP optical settings (excitation 488 nm/emission 520–540 nm).

### Pull-down assay

The complete coding sequences of *SlCV* and *SlBAM3* were PCR-amplified and subsequently inserted into pGEX-4 T-1 and pET-32a expression vectors and expressed in *Escherichia coli* BL21 using IPTG induction. Protein purification was performed by incubating the lysates for 4 h in binding buffer (50 mM Tris–HCl pH 7.5, 100 mM NaCl, 0.25% Triton X-100, 35 mM *β*-mercaptoethanol), followed by affinity chromatography using GST-tag Purification Resin (Beyotime, China) with 2 h rotation at 4°C as described by Lu et al. [18]. Non-specific proteins were removed through wash buffer treatment, and target protein detection was performed by immunoblot analysis using HIS- and GST-specific antibodies (Solarbio, China).

### Cell-free degradation assays *in vitro*

To examine protein stability, LNT-exposed leaves were snap-frozen and homogenized for protein extraction. Following BCA quantification, degradation assays were conducted by incubating SlBAM3-HIS with plant extracts in reaction buffer (25 mM Tris–HCl pH 7.5, 10 mM NaCl/MgCl₂, 5 mM DTT, 10 mM ATP) at 30°C. Time-course samples were analyzed by immunoblotting with anti-HIS antibody, normalized against Actin controls (Sangon Biotech, China).

### Statistical analysis

The experimental design followed a completely randomized approach. Statistical analysis was performed using SPSS version 22 (IBM SPSS STATISTICS, USA), with a significance threshold set at *P* < 0.05. Data visualization was conducted using Origin 2022 software (Origin Lab, Northampton, MA, USA). All primer sequences for vector construction are provided in Supplementary Table S2.

### Accession numbers

Sequence data from this article can be obtained from the Sol Genomics databases (https://solgenomics.net/) under the accession numbers listed in Supplementary Table S1.

## Supplementary Material

Web_Material_uhaf233

## Data Availability

The authors confirm that data from this study are available.
