# Supplementary material for: SlCV affects starch metabolism by regulating SlBAM3 stability under low night temperature stress in tomatoes
Source: Hortic Res. 2025 Sep 3;12(12):uhaf233. doi: 10.1093/hr/uhaf233 (PMC12682071; doi:10.1093/hr/uhaf233)
Supplement: Web_Material_uhaf233 [file web_material_uhaf233.zip › Supplemental Figures (Revision).docx]

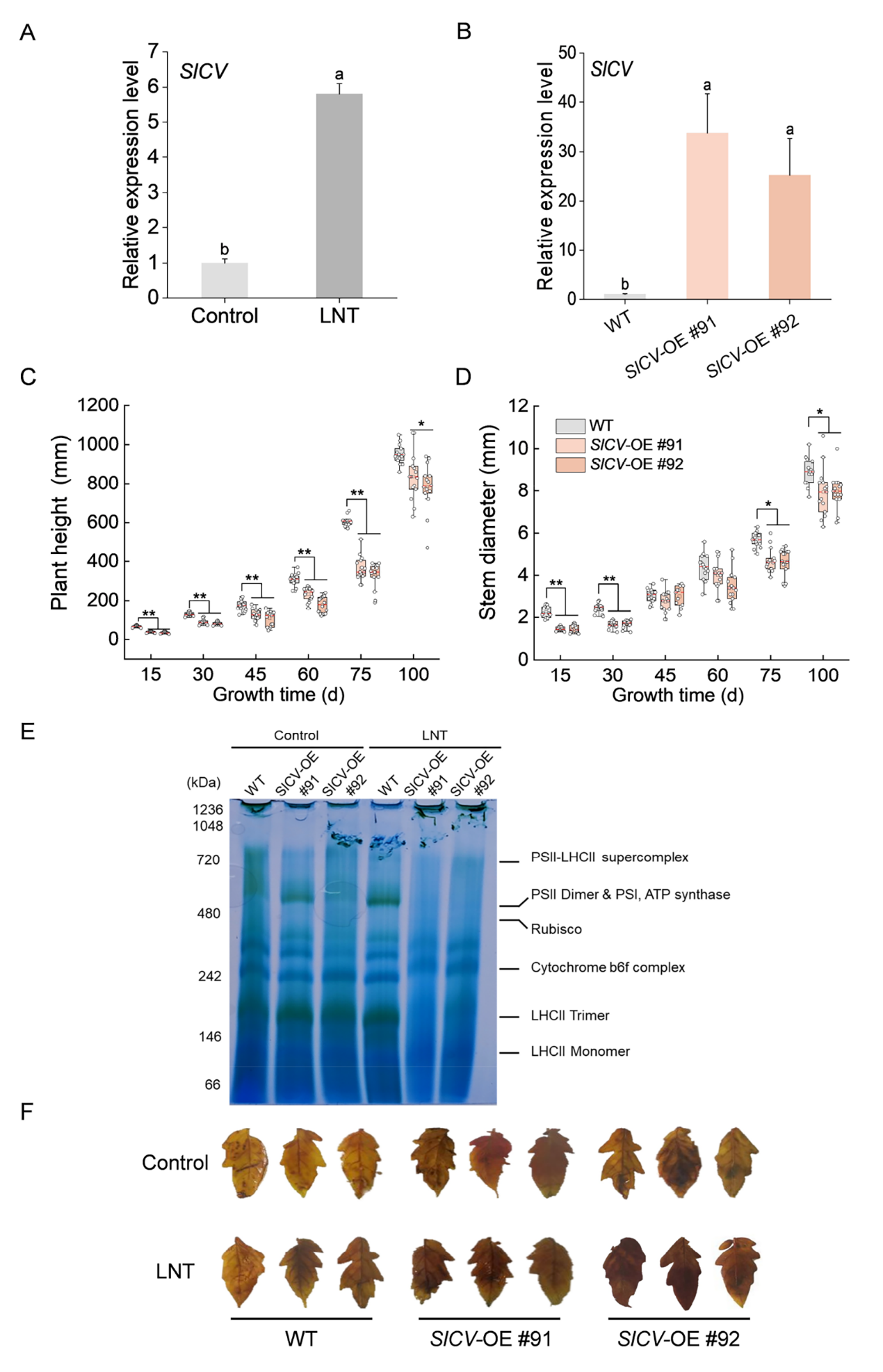


**Figure S1.** A) The relative expression level of *SlCV* under LNT stress. B) The relative expression level of *SlCV* in WT and *SlCV*-OE plants. C) Plant height and stem diameter D) of *SlCV*-OE plants at different growth stages. (**p* < 0.05, ***p* < 0.01). E) Blue-native PAGE of WT and *SlCV*-OE plants under control and LNT stress conditions. F) I/KI staining analysis of tomato leaves. At least three biological replicates were analyzed, with standard errors shown by vertical bars. Differences among treatments were analyzed by the one-way ANOVA comparison test (*p* < 0.05). Different letters indicate significant differences among treatments.


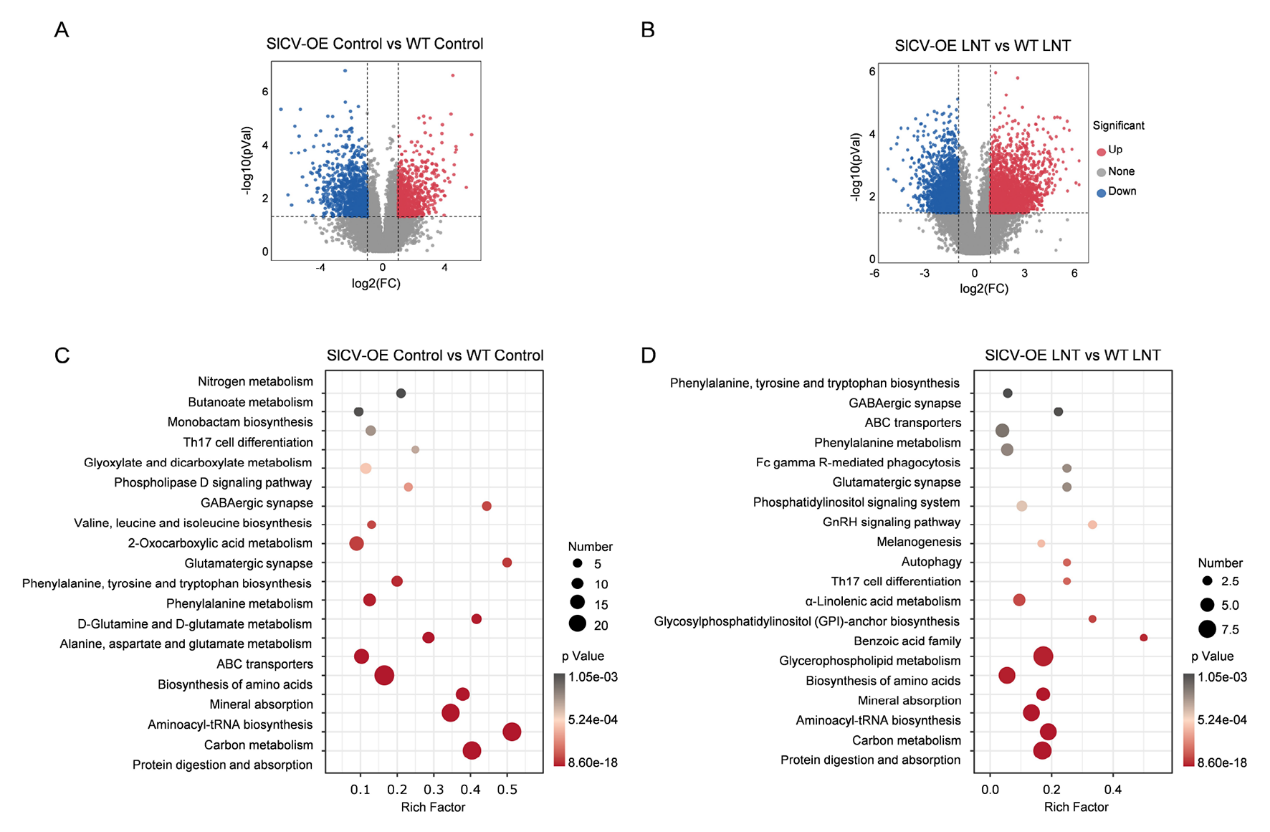


**Figure S2.** Volcano plot of differential metabolites between WT and *SlCV*-OE plants under control (A) and LNT stress conditions (B). Kyoto Encyclopedia of Genes and Genomes (KEGG) enrichment analysis of differential metabolites in WT and *SlCV*-OE plants under control (C) and LNT stress conditions (D).


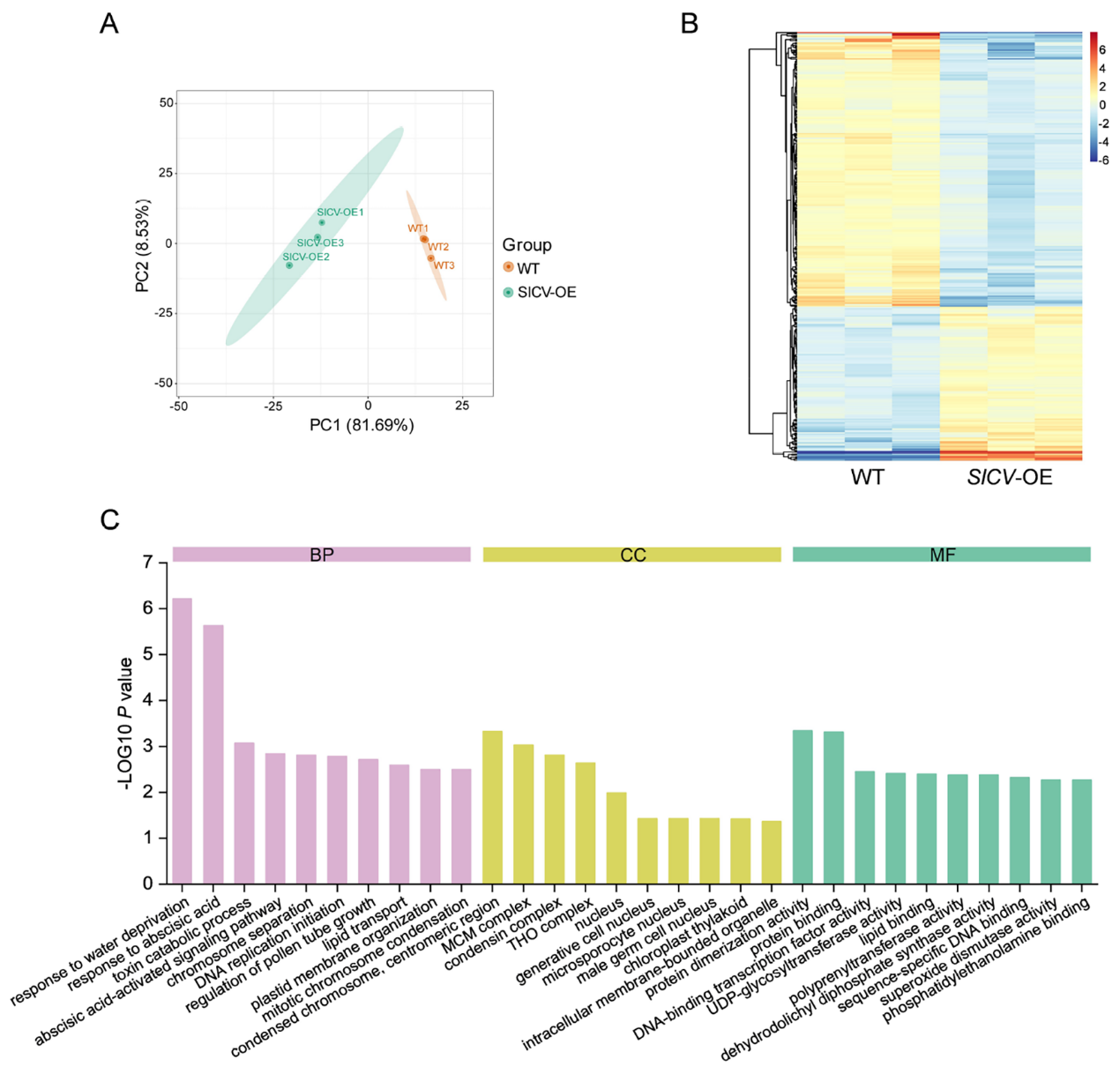
**Figure S3.** A) Principal component analysis (PCA) of differentially expressed genes (DEGs) in WT and *SlCV*-OE plants. B) Heatmap of differentially expressed genes between WT and SlCV-OE plants. C) Gene Ontology (GO) enrichment analysis of differentially expressed genes between WT and SlCV-OE plants. GO, Gene Ontology; BP, biological processes; CC, cellular components; MF, molecular functions.


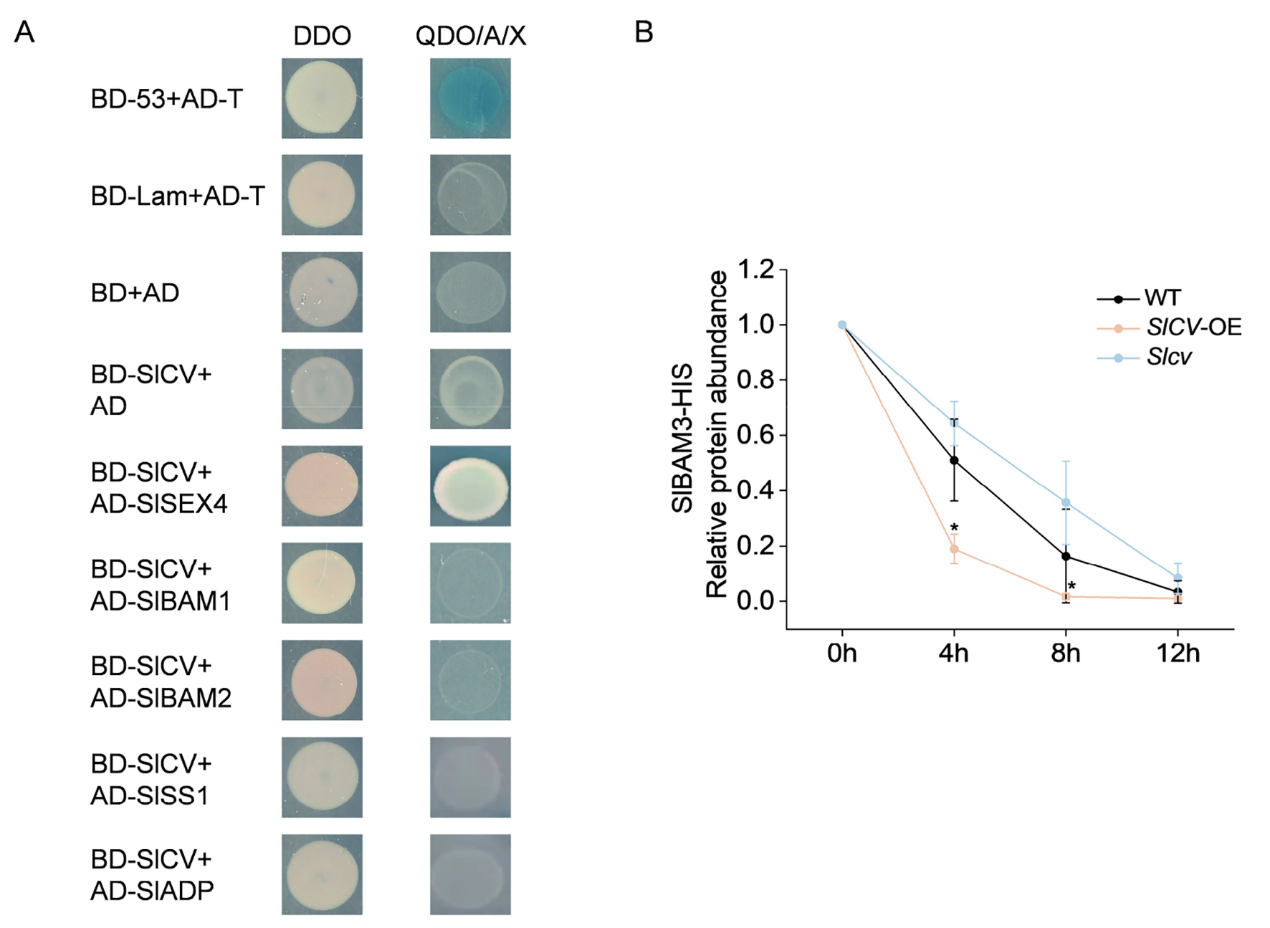


**Figure S4.** A) Yeast two-hybrid (Y2H) assays between SlCV and key proteins in starch metabolism. DDO, SD medium lacking Trp/Leu; QDO/A/X, SD medium lacking Trp/Leu/His/Ade and containing X-a-gal and aureobasidin A. The empty plasmids were used as controls. Blue color indicates protein interaction. B) The protein abundance of SlBAM-HIS in the cell-free degradation assay. The SlBAM3-HIS recombinant protein purified from Escherichia coli was co-incubated with the total protein extracts from leaves of WT, SlCV-OE, and Slcv plants treated with LNT stress. Immunoblot analysis was performed using anti-HIS antibody at different time points. The gray values were quantified using Image software, the three biological replicates were analyzed, with standard errors shown by vertical bars (* *p* < 0.05).


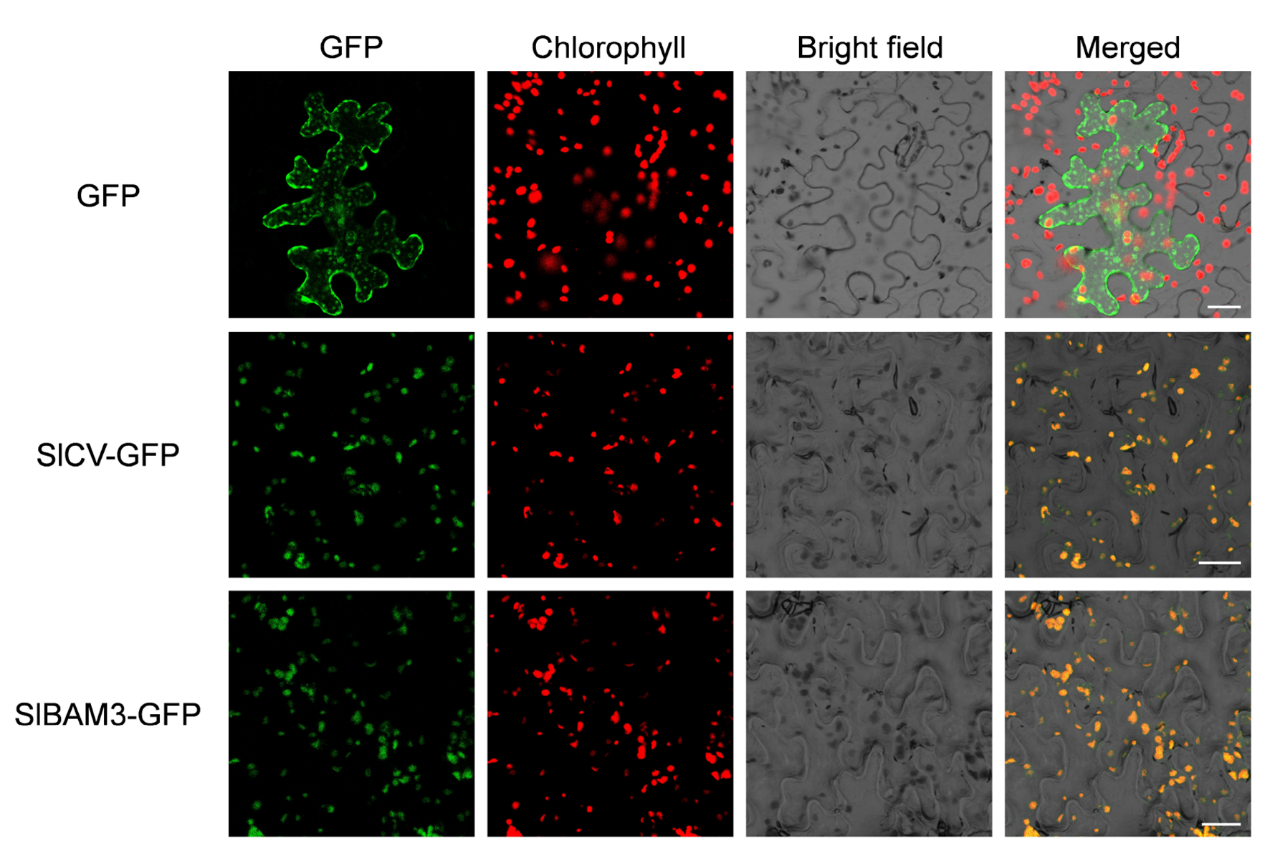


**Figure S5.** Subcellular localization of SlBAM3 (bar, 25 μm).


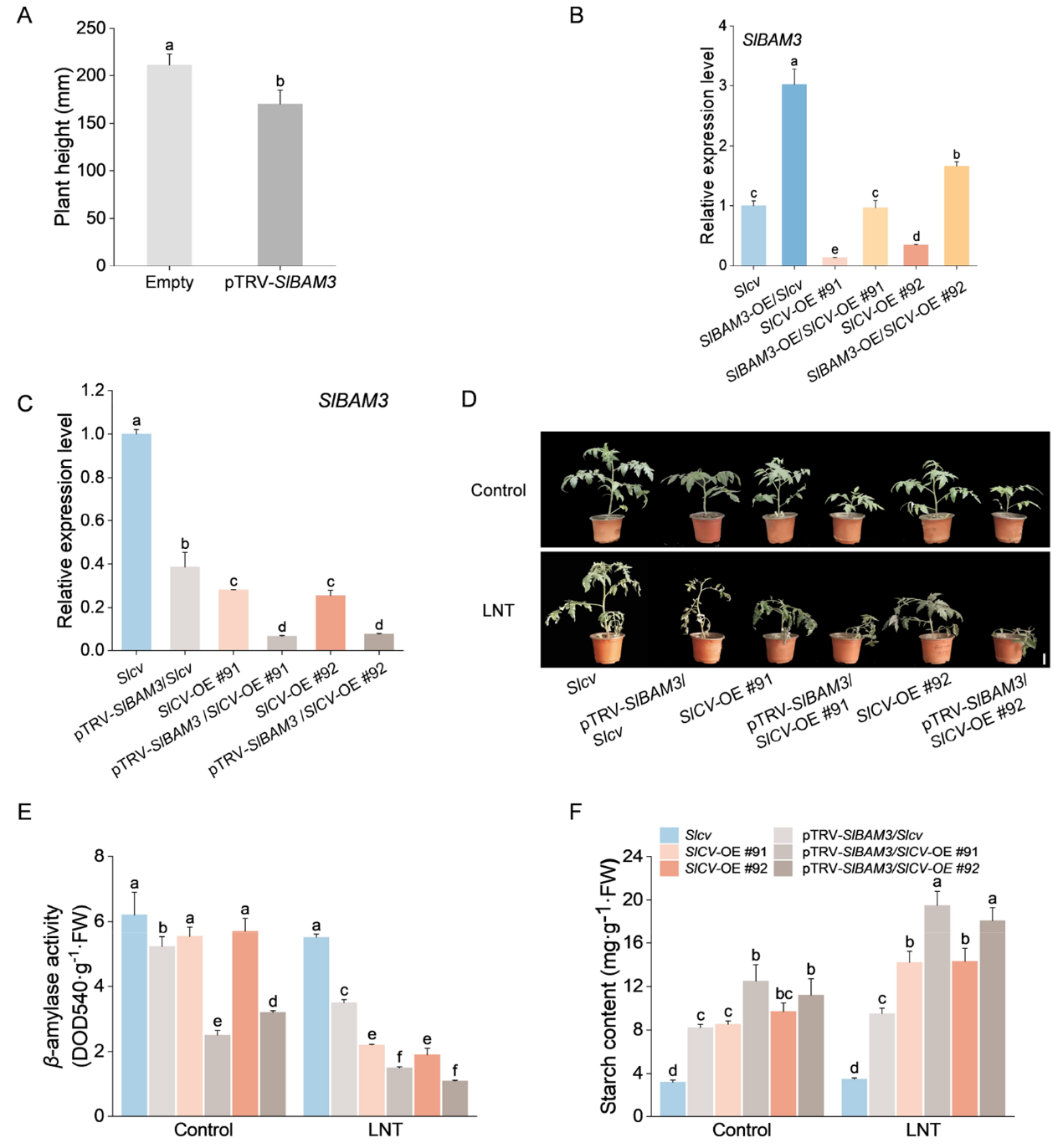
**Figure S6.** A) The plant height of WT (pTRV) and pTRV-*SlBAM3* (one-way ANOVA). B) Relative expression of SlBAM3 in *SlBAM3-*transient overexpression in *Slcv* mutant (*SlBAM3*-OE/*Slcv*) or SlCV-OE lines (*SlBAM3*-OE/*SlCV-*OE #91, *SlBAM3*-OE/*SlCV-*OE #92) plants (one-way ANOVA). C) Relative expression of SlBAM3 in *SlBAM3-*silenced in *Slcv* mutant (pTRV-*SlBAM3*/*Slcv*) or SlCV-OE lines (pTRV-*SlBAM3*/*SlCV-*OE #91, pTRV-*SlBAM3*/*SlCV-*OE #92) plants (one-way ANOVA). D) The phenotypes of *SlBAM3-*silenced in *Slcv* mutant (pTRV-*SlBAM3*/*Slcv*) or SlCV-OE lines (pTRV-*SlBAM3*/*SlCV-*OE #91, pTRV-*SlBAM3*/*SlCV-*OE #92) plants before and after LNT treatment (bar, 5 cm). E and F) The *β*-amylase activity of tomato leaves (two-way ANOVA, treatment *p* < 0.001, genotype *p* < 0.001, interaction *p* < 0.001). F) The starch content of tomato leaves (two-way ANOVA, treatment *p* < 0.001, genotype *p* < 0.001, interaction *p* < 0.001). At least three biological replicates were analyzed, with standard errors shown by vertical bars. Different letters indicate significant differences among treatments (*p* < 0.05).
