# Supplementary material for: SlCV affects starch metabolism by regulating SlBAM3 stability under low night temperature stress in tomatoes
Source: Hortic Res. 2025 Sep 3;12(12):uhaf233. doi: 10.1093/hr/uhaf233 (PMC12682071; doi:10.1093/hr/uhaf233)
Supplement: Web_Material_uhaf233 [file web_material_uhaf233.zip › Supplemental table S1.docx]

Supplemental Table S1 List of primer sequences used for qRT-PCR analysis

| **Gene** | **Accession number** | **Primer sequences (5’-3’)** |
| --- | --- | --- |
| *SlCV* | Solyc08g067630 | F-TCCCCTCAACCTCCTCCTACT |
|  |  | R-TGGGCATGATCTTTTTTCACTC |
| *SlBAM2* | Solyc08g005780 | F-CCGGATCCCCAATTTCCCAA |
|  |  | R-GGGATTGGATTGGGGCTGAA |
| *SlSEX4-2* | Solyc12g089210 | F-TGCTCGTGTTCTTTCTCGCT |
|  |  | R-AAACCTTTGGGACAGGCACA |
| *SlADP-2* | Solyc12g011120 | F-TTGCTGCACTTCCGATGGAT |
|  |  | R-GTGACAGTTCTTGCCGATGC |
| *SlSS2-3* | Solyc01g016660 | F-TGACCCAGTAGTAGGTGAGCA |
|  |  | R-TCAAGTTCCTTCTGCAATGCC |
| *SlBAM4* | Solyc01g094580 | F-GGTTCGTTGAAAGGTGTGCC |
|  |  | R-AGGATACCTCCACCCATGCT |
| *SlBAM3-1* | Solyc08g007130 | F-ACACAGCGCGAATTGTTCAC |
|  |  | R-CGGGCTCGAACAACCTTTTG |
| *SlADP-3* | Solyc07g019440 | F-GGTGGTGTTGGAACTCGTCT |
|  |  | R-GCAAGGTGACGATTGAGGGA |
| *SlSEX4-1* | Solyc03g097830 | F-CAGCAACAGCAGCACCAAC |
|  |  | R-GGTGCTTGTTGCTGTTCCTT |
| *SlBAM3-2* | Solyc08g077530 | F-TCTTTTGCACAAGCCAAGCC |
|  |  | R-CTCCACAAGGACCCATTCCC |
| *SlSS4* | Solyc02g071040 | F-TGTTGCGAAGGTAGGTGGTC |
|  |  | R-CCAGGGTGCTGAGGTTCAAT |
| *SlSEX1* | Solyc05g005020 | F-GCAAGGCTCAGGACAGACTT |
|  |  | R-ATGGTACCACTCTGCCTTGC |
| *SlSS3-1* | Solyc02g080570 | F-TAGAGAAGCCGCTATGCGTG |
|  |  | R-GCCATTTTCAGCAGGCAACA |
| *SlCBSS* | Solyc04g005080 | F-GGTGCTGGTGCTATTGCTTC |
|  |  | R-CCAACACCAACACCTTCACC |
| *SlGWD* | Solyc01g099600 | F-GGTGCTGGTGTTGCTACTGT |
|  |  | R-CCAACACCTTCACCTTCACC |
| *SlSS2-1* | Solyc02g088000 | F-GCCATGCAACAGGACCTACT |
|  |  | R-CACACGGTTCCCTCCGTAAA |
| *SlSS3-2* | Solyc07g042830 | F-CACTCATGCGATCAAGCACG |
|  |  | R-AGCATATGCGACAGCTCCTC |
| *SlSS1* | Solyc09g065510 | F-CTGCTACTGCTGGTGCTGTG |
|  |  | R-GTTCCTTCCACCAACACCTT |
| *SlADP-1* | Solyc07g056140 | F-CCGGAGACAAGTTGATGCCT |
|  |  | R-GCTCTCCTTGCGGTTTCTCT |
| *SlBAM1* | Solyc07g052695 | F-TTACCTTCCCATCGCCCAAATGC |
|  |  | R-CGCCACCTGCCTAACCAACTTC |
| *SlLSF1* | Solyc12g062250 | F-TCGGCAGTTAAACGACGACA |
|  |  | R-CACAGCAGTGATCCCCACAT |
| *SlSS2-2* | Solyc07g025180 | F-GACCCAGTAGTAGGTGAGCA |
|  |  | R-GTCCAAGGAGCAGTTCAGGT |
| *ACTIN* | Solyc11g005330 | F-TGTCCCTATTTACGAGGGTTATGC  R-CAGTTAAATCACGACCAGCAAGAT |
|  |  |  |
