# Supplementary material for: SlCV affects starch metabolism by regulating SlBAM3 stability under low night temperature stress in tomatoes
Source: Hortic Res. 2025 Sep 3;12(12):uhaf233. doi: 10.1093/hr/uhaf233 (PMC12682071; doi:10.1093/hr/uhaf233)
Supplement: Web_Material_uhaf233 [file web_material_uhaf233.zip › Supplemental table S2 (Revision).docx]

Supplemental Table S2 PCR primer sequences used for vector construction

| Primer | Primer sequence（5'-3'） |
| --- | --- |
| SlCV-BD-F | ATGGCCATGGAGGCCGAATTC ATGGCTATTTCAACAAAGTTCTGC |
| SlCV-BD-R | TAGTTATGCGGCCGCTGCAGG TCACATAGTGAAACATCCTTTACT |
| SlCV-nLUC-F | CGAGCTCGGTACCCGGGATCC ATGGCTATTTCAACAAAGTTCTGC |
| SlCV-nLUC-R | CGCGTACGAGATCTGGTCGAC CATAGTGAAACATCCTTTACTAAAT |
| SlCV-nYFP-F | CCCAGGCCTACTAGTGGATCC ATGGCTATTTCAACAAAGTTCTGC |
| SlCV-nYFP-R | GGGAAATTCGAGCTCCTACCC TCACATAGTGAAACATCCTTTACT |
| SlCV-GST-F | TTCCAGGGGCCCCTGGGATCC ATGGCTATTTCAACAAAGTTCTGC |
| SlCV-GST-R | GTCACGATGCGGCCGCTCGAG TCACATAGTGAAACATCCTTTACT |
| SlCV-GFP-F | GAGCTCGGTACCCGGGGATCC ATGGCTATTTCAACAAAGTTCTGC |
| SlCV-GFP-R | GCCCTTGCTCACCATGTCGAC CATAGTGAAACATCCTTTACTAAAT |
| SlBAM3-cLUC-F | ccggggcGGTACCCGGGAT ATGGCTTTAACACCTTGTTCTT |
| SlBAM3-cLUC-R | ATACGAACGAAAGCTCTGCAG CTACACAACTGCAACCTCTG |
| SlBAM3-pTRV2-F | GTGAGTAAGGTTACCGAATTC ATGGCTTTAACACCTTGTTCTT |
| SlBAM3-PTRV2-R | CGTGAGCTCGGTACCGGATCC CTTGTTCATATTGCCACCAAT |
| SlBAM3-HIS-F | gctgatatcggatccgaattc ATGGCTTTAACACCTTGTTCTT |
| SlBAM3-HIS-R | ctcgagtgcggccgcaagcttg CACAACTGCAACCTCTGTAG |
| SlBAM3-PRI101-F | tcttcactgttgatacatatg ATGGCTTTAACACCTTGTTCTT |
| SlBAM3-PRI101-R | agagttgttgattcagaattc CTACACAACTGCAACCTCTG |
| SlBAM3-cYFP-F | Tggcgcgccactagtggatcc ATGGCTTTAACACCTTGTTCTT |
| SlBAM3-cYFP-R | Aacatcgtatgggtacatccc CACAACTGCAACCTCTGTAG |
| SlBAM3-GFP-F | GAGCTCGGTACCCGGGGATCC ATGGCTTTAACACCTTGTTCTT |
| SlBAM3-GFP-R | GCCCTTGCTCACCATGTCGAC CACAACTGCAACCTCTGTAG |
| SlBAM3-AD-F | GCCATGGAGGCCAGTGAATTC TGGCTTTAACACCTTGTTCTT |
| SlBAM3-AD-R | CAGCTCGAGCTCGATGGATCC CTACACAACTGCAACCTCTG |
| SlSEX4-AD-F | GCCATGGAGGCCAGTGAATTC ATGGCCGCCATTTTCTCTCAG |
| SlSEX4-AD-R | CAGCTCGAGCTCGATGGATCC TTACAAGCCAAGAACATGAAGCATT |
| SlBAM1-AD-F | GCCATGGAGGCCAGTGAATTC ATGAATATCAGTACGTCTAGC |
| SlBAM1-AD-R | CAGCTCGAGCTCGATGGATCC TCAGTTAGATAGAAAGAACTTGTT |
| SlSS1-AD-F | GCCATGGAGGCCAGTGAATTC ATGAATAATGGTTATAGCAATCTT |
| SlSS1-AD-R | CAGCTCGAGCTCGATGGATCC TTACGAGTCTGTAGTGTGTC |
